# New alternative ingredients and genetic selection are the next game changers in rainbow trout nutrition: a metabolomics appraisal

**DOI:** 10.1038/s41598-023-46809-2

**Published:** 2023-11-10

**Authors:** Simon Roques, Catherine Deborde, Sandrine Skiba-Cassy, Françoise Médale, Mathilde Dupont-Nivet, Florence Lefevre, Jérome Bugeon, Laurent Labbé, Yann Marchand, Annick Moing, Benoit Fauconneau

**Affiliations:** 1 INRAE, Univ. Pau & Pays Adour, E2S UPPA, Nutrition, Métabolisme et Aquaculture, UMR 1419, 64310 Saint Pée sur Nivelle, France; 2 Phileo by Lesaffre, 59700 Marcq-en-Barœul, France; 3 Bordeaux Metabolome, MetaboHUB, Centre INRAE de Nouvelle-Aquitaine Bordeaux, 33140 Villenave d’Ornon, France; 4 Centre INRAE de Nouvelle-Aquitaine Bordeaux, INRAE, Univ. Bordeaux, Biologie du Fruit et Pathologie, UMR 1332, 33140 Villenave d’Ornon, France; 5 Université Paris-Saclay, INRAE, AgroParisTech, Génétique Animale et Biologie Intégrative, UMR 1313, 78350 Jouy-en-Josas, France; 6 INRAE, Laboratoire de Physiologie et Génomique des Poissons, UR 1037, 35000 Rennes, France; 7 INRAE, UE0937, PEIMA, 29450 Sizun, France; 8 Le Gouessant, 22402 Lamballe, France; 9 Present Address: Université Clermont Auvergne, INRAE, VetAgro Sup, UMR Herbivores, 63122 Saint-Genes-Champanelle, France; 10 Present Address: INRAE, Biopolymères Interactions Assemblages, UR1268, 44300 Nantes, France; 11 Present Address: INRAE, BIBS Facility, Centre INRAE Pays de Loire – Nantes, 44000 Nantes, France

**Keywords:** Physiology, Zoology

## Abstract

The formulation of sustainable fish feeds based on plant ingredients supplemented by alternative ingredients to plant (insect, micro-algae, yeast) and genetic selection of fish for plant-based diets were tested on rainbow trout in two separate experiments. Plant-based diets and corresponding diets supplemented with an ingredient mix: insect, micro-algae and yeast in Experiment A, and insect and yeast in Experiment B were compared to commercial-like diets. In experiment A, the mix-supplemented diet was successful in compensating the altered growth performance of fish fed their respective plant-based diet compared to those fed the commercial diet, by restoring feed conversion. In experiment B, the selected line demonstrated improved growth performances of fish fed mix-supplemented and plant-based diets compared to the non-selected line. Metabolomics demonstrated a plasma compositional stability in fish fed mix-supplemented and basal plant-based diets comprising an amino acid accumulation and a glucose depletion, compared to those fed commercial diets. The selected line fed mix-supplemented and commercial diets showed changes in inositol, ethanol and methanol compared to the non-selected line, suggesting an involvement of microbiota. Changes in plasma glycine-betaine content in fish fed the mix-supplemented diet suggest the ability of the selected line to adapt to alternative ingredients.

## Introduction

Combined with responsible fishing policies, the increasing demand for aquatic products challenges the development of sustainable and efficient fish feed for aquaculture, which currently provides about 50% of the aquatic products consumed^1^. Plant ingredients make up at least 90% of the fish feed used for many fish species, including carnivorous ones such as the rainbow trout^2,3^. However, sustainable fish feeds free of marine ingredients are not yet sufficiently performant to guarantee a fair economic income to farmers. Fish feeds can be improved by selecting high-quality plant proteins, or by including alternative ingredients of plants ingredients and fish products, that meet the nutritional requirements of fish, increase farming profitability, and support environmental preservation and consumer expectations. A substantial research effort has been conducted to assess the efficacy of a wide range of alternative ingredients to plant-based feedstuffs, from single-cell ingredients to animal by-products.

Single-cell ingredients, such as micro-algae and yeast used as supplements in plant-based fish feeds, satisfy protein and lipid requirements of carnivorous fish^4–7^. Some of these microorganisms also contribute to restore the digestive and microbiota functions altered by plant-based diets^5^. On one hand, the production of bacteria and yeast has been optimised and can be implemented at a large scale. On the other hand, most of the micro-algae productions are still at the pilot scale on a few taxa collected from wild stock^8^. Alternative ingredients based on animal by-products tend to mimic natural fish food. This is the case, for example, of terrestrial insects^9,10^, whose larvae are beginning to be produced at a pilot scale and even at an industrial scale. These ingredients contain substantial protein levels and are well adapted to fish amino acid requirements^5^. They also contain specific functional compounds relevant for feed acceptability, gut integrity preservation, and metabolism. The incorporation of most of these alternative ingredients in fish feed has only been tested on individual ingredients; however, combining them could better fulfil fish nutritional requirements through their complementarity.

Genetically selecting fish is a complementary strategy to adapt fish to these feeds free of fish ingredients. This strategy is based on selecting fish that display the highest performances when fed plant-based diets over several generations. There is indeed a genetic basis for the adaptation to plant-based diets that has been demonstrated in various fish species^11,12^. Although farmed fish are likely to be subjected to natural selection, as commercial feeds now contain high levels of plant-based feedstuff^13^, continuous selection over generations has refined selected lines that outperform these non-selected lines when fed plant-based diets^14–17^.

The development of metabolomic approaches to fish nutrition^18,19^ allows us to create a comprehensive view of the metabolic syndrome of fish fed plant-based diets, previously described through transcriptomics and proteomics. These more recent approaches demonstrate the combined alteration of protein, lipid and carbohydrate metabolisms induced by plant-based diets in specific pathways^20–23^, including in selected lines^16,24–26^. Metabolomic approaches link the disruption of energy and protein metabolisms to the asynchronous absorption of essential nutrients in fish fed plant-based diets^27^, which can be restored by supplementing fish feeds with alternative ingredients^28–30^. Essential liver functions, such as phospholipid synthesis and one-carbon metabolism, are also affected, as demonstrated for instance by the status of betaine and related metabolites^28,29,31–33^. Moreover, specific metabolites (histidine, taurine, betaine) account for protective adaptations to preserve cell vital functions such as osmolality and buffering capacity^34^. All these changes constitute the metabolic scope of fish fed plant-based diets. Genetic selection for adaptation to a plant-based diet can compensate for digestive alterations^35,36^ and the related protein metabolic disturbances^17^ induced by plant-based diets. Further investigations using metabolomics and genomic approaches have shown other beneficial outcomes of this selection on various metabolic pathways^16,24,35^. However, a coherent metabolic scheme has not yet emerged, and this could be partly related to the existence of the different lines selected for adaptation to plant-based diets^36^. These dietary and/or selection-induced metabolic changes are informative on the mechanisms that lead to modifications in the performances of fish fed plant-based diets. However, the metabolic consequences of combining supplementation with a mix of alternative ingredients to plant-based ingredients with trout line selection are still unknown.

The purpose of this study was to test the relevance of using a mix of alternative ingredients, namely yeast, micro-algae and insects, in selected and non-selected trout lines, in order to restore fish performance and metabolism that have been altered by a plant-based diet. In a first step, two mix-supplemented diets were provided to a non-selected line and compared to their respective non-supplemented plant-based diets that differed in the level of protein purification of plant ingredients. These four diets were then compared to a commercial diet composed of marine ingredients. In a second step, the mix-supplemented diets, fine-tuned from the previous ones, were tested on a line selected for a plant-based diet as well as on a control non-selected line. We used rainbow trout, one of the first fish species reared in Europe, as a model species for salmonids. This species benefits from solid background data on long-term plant-based diet feeding throughout the life cycle of the fish^37^ and the existence of selected lines that have adapted to plant-based diets^15,16,36^.

## Results

The growth performances and plasma metabolome were analysed in rainbow trout fed either plant-based diets (PB) or corresponding plant-based diets supplemented with a mix of alternative ingredients, insect (I), spirulina (S) and yeast (Y), compared to commercial-like diets comprising marine ingredients (COM) in two separate experiments (Fig. S1). In a first experiment (Experiment A), a set of diets (PB1, PB2, ISY1, ISY2, COM-A) was tested in a non-selected line, and in a second experiment (Experiment B), another set of diets (PB3, IY3, COM-B) was tested in a selected line adapted to plant-based diet compared to a non-selected one (Fig. S2). The feed formulations in the two experiments are given respectively in Tables 1 and 2. The comparison of the feed formulation between the two experiments as well as the amino acid composition and the fibre contents of the experimental diets are given in Tables S1–S3.

**Table 1:** Formulation and proximate composition of commercial (COM-A), plant-based (PB1 & PB2) and plant-based experimental feeds supplemented with insect hydrolysate, spirulina and yeast extract (ISY1 & ISY2) for rainbow trout feeding in Experiment A.

Ingredients (g.100g^–1^ FW)	COMA	PB1	PB2	ISY1	ISY2
Fish meal	21.03				
Fish oil	4.88				
DHA-rich algae meal		6.84	6.84	6.84	6.84
Insect hydrolysate				5.00	5.00
Spirulina				5.00	5.00
Yeast extract				5.00	5.00
Processed animal proteins^1^	15.00				
Vegetal oils^2^	14.65	18.10	17.75	16.75	16.50
Plant proteins^3^	42.7	70.60	70.53	57.14	56.95
Rapeseed lecithin		1.00	1.00	1.00	1.00
Monocalcium phosphate		1.20	1.25	1.05	1.10
Phytase		0.02	0.02	0.02	0.02
Lysine 78%	0.39	0.50	1.00	0.50	1.00
DL-methionine 98%	0.44	0.65	0.52	0.61	0.50
Threonine 98%	0.20	0.20	0.20	0.20	0.20
Vitamin premix^4^	0.25	0.30	0.30	0.30	0.30
Vitamin C monophosphate 35 %	0.04	0.04	0.04	0.04	0.04
Mineral premix^5^	0.25	0.30	0.30	0.30	0.30
Liquid choline	0.15	0.25	0.25	0.25	0.25
Antioxidant	0.10	0.10	0.10	0.10	0.10
Antifungal	0.15	0.15	0.15	0.15	0.15
Proximate composition^6^					
Dry matter (% FW)	95.8	96.9	94.6	96.9	96.5
Proteins (% DM)	43.0	44.7	45.7	45.0	45.3
Lipids (% DM)	21.0	21.6	21.4	21.2	21.4
Ash (% DM)	7.3	5.4	5.4	6.3	5.9
Energy (kJ.g^–1^ DM)	24.1	24.0	24.4	24.0	24.4

^1^Processed animal protein (feather meal protein, blood product, poultry meal, 5/3/7 g.kg^–1^).

^2^Vegetable oils: rapeseed oil and linseed oil.

^3^Plant proteins: COM: soy concentrate, soybean meal, rapeseed meal, peeled fava bean and wheat. PB1: wheat gluten, hydrolysed wheat gluten, pea protein, fava bean protein concentrate, soy concentrate, wheat. PB2: pea protein, corn gluten, soybean meal, guar meal, peeled fava bean. ISY1: a fraction of wheat gluten, pea protein, fava bean protein, soy concentrate and wheat as well as a fraction of rapeseed oil and linseed oil were substituted by the insect-spirulina-yeast fraction. ISY2: a fraction of pea protein, corn gluten, soybean meal, guar meal, peeled fava bean and wheat as well as a fraction of rapeseed oil and linseed oil were substituted by the insect-spirulina-yeast fraction.

^4^Vitamin premix composition: vitamin A (retinyl acetate/3a672a) 4 000 000 UI/kg; vitamin D (cholecalciferol/3a671) 700 000 UI/kg; vitamin E (alpha-tocopheryl acetate/3a700) 80 000 UI/kg; vitamin K3 (menadione/3a711) 4 g/kg; vitamin B1 (thiamine mononitrate/3a821) 4 g/kg; vitamin B2 (riboflavin) 6 g/kg; vitamin B6 (pyridoxine hydrochloride/3a831) 6 g/kg; vitamin B12 (cyanocobalamin) 20 mg/kg; vitamin B5 (D-calcium pantothenate/3a841) 12 g/kg; nicotinic acid (vitamin PP—B3—niacin 3a314/niacinamide 3a315) 12 g/kg; folic acid (vitamin B9/3a316) 3.6 g/kg; biotin (vitamin B8/3a880) 0.4 g/kg.

^5^Mineral premix composition: iodine (calcium iodide anhydrous/3b202) 0.4 g/kg; manganese (manganese oxide II/3b502) 20 g/kg; zinc (zinc oxide/3b603); 40 g/kg; iron (iron II sulfate monohydrate/3b103) 32 g/kg; copper (copper II sulfate pentahydrate/3b405) 1.2 g/kg.

^6^*FW* fresh weight, *DM* dry matter.

**Table 2:** Formulation and proximate composition of commercial (COM-B), plant-based (PB3) and plant-based experimental feeds supplemented with insect hydrolysate and yeast extract (IY3) for rainbow trout feeding in Experiment B.

Ingredients (g.100g^–1^ FW)	COM-B	PB3	IY3
Fish meal	25.63		
Fish oil	6.00		
DHA-rich algae meal		5.40	5.40
Insect hydrolysate			5.00
Yeast extract proteins			5.00
Vegetal oils^1^	13.00	16.60	15.80
Plant proteins^2^	53.00	72.50	64.00
Rapeseed lecithin		1.00	1.00
Monocalcium phosphate	1.00	1.65	1.62
Phytase	0.02	0.02	0.02
Lysine 78%	0.43	0.96	0.73
DL-methionine 98%	0.30	0.50	0.50
Threonine 98%	0.20	0.20	0.20
Vitamin premix^3^	0.25	0.30	0.30
Vitamin C monophosphate 35 %	0.04	0.04	0.04
Mineral premix^4^	0.25	0.30	0.30
Liquid choline	0.15	0.25	0.25
Antioxidant	0.15	0.15	0.15
Antifungal	0.15	0.15	0.15
Astaxanthin 10%	0.03	0.03	0.03
Proximate composition^5^			
Dry matter (% FW)	93.2	92.1	91.9
Proteins (% DM)	43.4	42.6	42.5
Lipids (% DM)	20.8	22.3	21.3
Ash (% DM)	8.0	5.2	5.3
Energy (kJ.g^–1^ DM)	24.1	24.8	24.9

^1^Vegetable oils: rapeseed oil and linseed oil.

^2^Plant proteins: COM: wheat gluten, corn gluten, soy concentrate, soybean meal, rapeseed meal, peeled fava bean and wheat. PB3: corn gluten, pea protein, soybean meal, guar meal, peeled fava bean, wheat. IY3: a fraction of wheat gluten, corn gluten, pea protein, soybean meal, guar meal and wheat as well as a fraction of rapeseed oil were substituted by insect hydrolysate & yeast extract fraction.

^3^Vitamin premix composition: vitamin A (retinyl acetate/3a672a) 4 000 000 UI/kg; vitamin D (cholecalciferol/3a671) 700 000 UI/kg; vitamin E (alpha-tocopheryl acetate/3a700) 80 000 UI/kg; vitamin K3 (menadione/3a711) 4 g/kg; vitamin B1 (thiamine mononitrate/3a821) 4 g/kg; vitamin B2 (riboflavin) 6 g/kg; vitamin B6 (pyridoxine hydrochloride/3a831) 6 g/kg; vitamin B12 (cyanocobalamin) 20 mg/kg; vitamin B5 (D-calcium pantothenate/3a841) 12 g/kg; nicotinic acid (vitamin PP—B3—niacin 3a314/niacinamide 3a315) 12 g/kg; folic acid (vitamin B9/3a316) 3.6 g/kg; biotin (vitamin B8/3a880) 0.4 g/kg.

^4^Mineral premix composition: iodine (calcium iodide anhydrous/3b202) 0.4 g/kg; manganese (manganese oxide II/3b502) 20 g/kg; zinc (zinc oxide/3b603); 40 g/kg; iron (iron II sulphate monohydrate/3b103) 32 g/kg; copper (copper II sulphate pentahydrate/3b405) 1.2 g/kg.

^5^*FW* fresh weight, *DM* dry matter.

### Growth performances

The growth performances and the somatic indices are presented in Table 3 for Experiment A and in Table 4 for Experiment B. In Experiment A, the final body weight of the fish fed the ISY2 diet was significantly higher (*P < 0.05*) than that of the fish fed the PB1 diet. Fish fed the other diets showed intermediate responses. The specific growth rate was not significantly different (ANOVA) between diets although the same pattern of differences in final body weight could be observed as the one noted between the ISY2 and PB1 diets. The voluntary feed intake and the feed conversion ratio were significantly higher in fish fed the PB1 diet than in fish fed the PB2 and ISY1 diets (*P < 0.05*), and the latter were significantly higher (*P < 0.05*) than in fish fed the commercial diet COM-A.

**Table 3:** Zootechnical performances of rainbow trout (initial body weight 49.1 g) fed two different plant-based diets (PB1 & PB2) for 84 days, supplemented with 5% insect hydrolysate, 5% spirulina and 5% yeast extract (ISY1 & ISY2), and a reference commercial-like diet (COM-A) in Experiment A.

	COM-A	PB1	PB2	ISY1	ISY2	ANOVA^3^
Final body weight^1^(g)	279.1^ab^ ± 16.9	247.1^b^ ± 14.0	288.5^ab^ ± 24.1	273.6^ab^± 14.0	295.6^a^ ± 8.5	F 3.7 P 5.10^–2^
Specific growth rate^1,2^ *(%.d*^*–1*^*)*	2.03 ± 0.02	1.93 ± 0.08	2.02 ± 0.16	2.04 ± 0.07	2.15 ± 0.06	F 2.0 P NS
Voluntary feed intake^1^ *(% body weight.d*^*–1*^*)*	1.15^c^ ± 0.02	1.32^a^ ± 0.02	1.24^b^ ± 0.02	1.23^b^ ± 0.03	1.20^bc^ ± 0.01	F 22.5 P 1.10^–4^
Feed conversion ratio^1^ *(g feed intake/g body weight growth))*	0.82^c^ ± 0.01	0.98^a^ ± 0.03	0.88^b^ ± 0.02	0.88^b^ ± 0.02	0.83^c^ ± 0.01	F 32.4 P 5.10^–5^

When ANOVA for differences between diets was significant, a multiple mean comparison Tukey’s test was applied. For each dependant variable, means with different subscript letters are significantly different (*P*<0.05).

^1^Mean ± SD of triplicate tanks for final body weight, growth, feed intake and efficiency ratios.

^2^Specific growth rate calculated as a ratio of (log of final body weight minus log of initial body weight) to the duration time (%. D^–1^).

^3^F value and *P*-value of one-way ANOVA; NS for *P *> 0.05.

**Table 4:** Zootechnical performances of rainbow trout lines (initial body weight 66.7 g) selected (SEL) or not-selected (nonSEL), fed a reference commercial-like diet (COM-B), a plant-based diet (PB3) or a mix-supplemented plant-based diet with 5% insect hydrolysate and 5% yeast cell wall (IY3) for 84 days in Experiment B.

Diet line	COM-B SEL	PB3 SEL	IY3 SEL	COM-B nonSEL	PB3 nonSEL	IY3 nonSEL	2-ANOVA diet effect^3^	2-ANOVA line effect	2-ANOVA interaction diet × line
Final body weight^1^ (g)	338.3^a^ ± 7.9	275.2^c^ ± 5.9	316.2^b^ ± 4.7	336.2^a^ ± 8.8	246.1^d^± 2.9	278.9^c^± 3.5	F 243 *P 2.10*^*–10*^	F 65 *P 4.10*^*–6*^	F 14 *P 7.10*^*–4*^
Specific growth rate^1,2^ *(%.d*^*–1*^*)*	1.88^a^ ± 0.03	1.61^d^ ± 0.05	1.79^b^ ± 0.01	1.94^a^± 0.01	1.57^d^± 0.02	1.74^c^± 0.00	F 217 *P 4.10*^*–10*^	F 0.5 *P NS*	F 7.5 *P 8.10*^*–3*^
Voluntary feed intake^1^ *(% body weight.d*^*–1*^*)*	1.71^a^ ± 0.00	1.51^d^ ± 0.01	1.67^b^ ± 0.00	1.71^a^± 0.01	1.42^e^± 0.01	1.57^c^± 0.01	F 1212 *P 1.10*^*–14*^	F 268 *P 1.10*^*–9*^	F 63 *P 5.10*^*–7*^
Feed conversion ratio^1^ *(g feed intake/g body weight growth))*	0.88^bc^ ± 0.02	0.90^ab^ ± 0.01	0.92^a^ ± 0.02	0.86^d^± 0.01	0.89^bc^± 0.01	0.89^c^± 0.00	F 1.3 *P NS*	F 14 *P 3.10*^*–3*^	F 0.8 *P NS*

When two-way analyse of variance (2-ANOVA) test for differences between diets or between lines were significant, a multiple mean comparison Tukey’s test was applied. For each dependant variable, means with different subscript letters are significantly different - (*P *< 0.05).

^1^Mean ± SD of triplicate tanks for final body weight, growth rate, feed intake and efficiency ratios.

^2^Specific growth rate calculated as a ratio of (log of final body weight minus log of initial body weight) to the duration time (%. D^–1^).

^3^F values and *P*-values of the two-way ANOVA, NS for *P *> 0.05.

In Experiment B (Table 4), the final body weight, the specific growth rate, and the voluntary feed intake of fish fed the IY3 supplemented diet were significantly higher (*P<0.05*) than those of fish fed the plant-based diet (PB3) but they were significantly lower than those of fish fed the COM-B diet when compared within each of the fish line. The feed conversion ratio of fish fed the IY3 supplemented diet was significantly higher than that of the COM-B but not significantly different from the PB3 diets within each of the fish line. When comparing the selected and the non-selected lines, we observed that the final body weight, the specific growth rate, and the voluntary feed intake were significantly higher in selected line than in non-selected line for fish fed IY3 and PB3 diets but such difference was not observed in COM-B diet. The feed conversion ratio of the selected line were significantly higher than that of the non selected line whatever the diet. However, we noted a highly significant interaction between line and diet in terms of final body weight (*P = 7.10*^*–4*^), specific growth rate (*P = 8.10*^*–3*^) and voluntary feed intake (*P = 5.10*^*–7*^). The selected line of fish fed the IY3 diet had a significantly higher final body weight and a significantly higher feed intake than the non-selected line fed the same diet, but such a difference was not observed in the fish that were fed the COM-B diet. The feed conversion ratio was significantly higher in fish of the selected line fed the supplemented plant-based diet IY3 than in those fed the two other diets while in non selected line the feed conversion ratio of the fish fed the COM-B diet was significantly higher than those fed the two other diets.

### Plasma ^1^H-NMR of fish fed the PB supplemented diet

**Figure 1:**
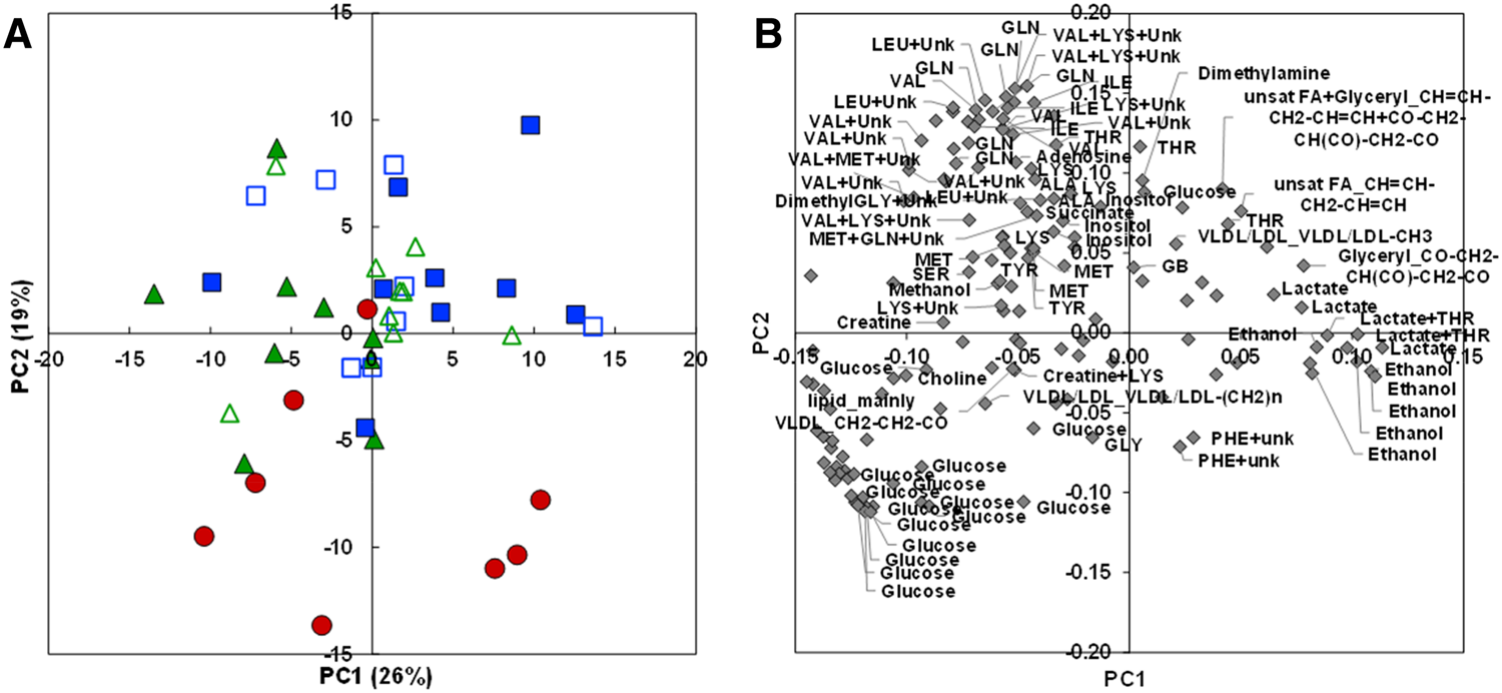
PCA of ^1^H-NMR metabolite profile in plasma of trout fed plant-based-diets (PB1 closed green triangles; PB2, open green triangles) mix-supplemented with insect, spirulina and yeast ingredients (ISY1 closed blue squares, ISY2 open blue squares) compared to a commercial-like diet (COM-A, closed red circles) in Experiment A. (**A**) Scores plot on the PC1 × PC2 plan. (**B**) Loadings plot. Identified buckets with an absolute value of loading higher than 0.05 are annotated.

In Experiment A, a PCA showed that the plasma NMR profiles of fish fed the PB1 and PB2 plant-based and the ISY1 and ISY2 supplemented plant-based diets were slightly discriminated compared to the fish fed the COM-A diet (Fig. 1A), but there was no discrimination between the PB1 or PB2 plant-based diets and their respective ISY1 or ISY2 supplemented diets. The loadings plot (Fig. 1B) showed an opposition between on one hand circulating lipids and final metabolites of energy metabolism -ethanol and lactate -and on the other hand circulating glucose and amino acids for the first principal component (PC1), but this was more related to individual differences than to the diet when compared to the scores plot (Fig. 1A). The differences between the COM-A and plant-based diets (PB and supplemented ISY) on the second principal component (PC2, 19 % of total variance) were related to glucose on the negative side, higher in fish fed the COM-A diet, and to essential amino acids (LEU, VAL, ILE, MET, THR), glutamine, dimethylamine and adenosine on the positive side, higher in fed the PB3 plant-based diet and the IY3 supplemented plant-based diets.

**Figure 2:**
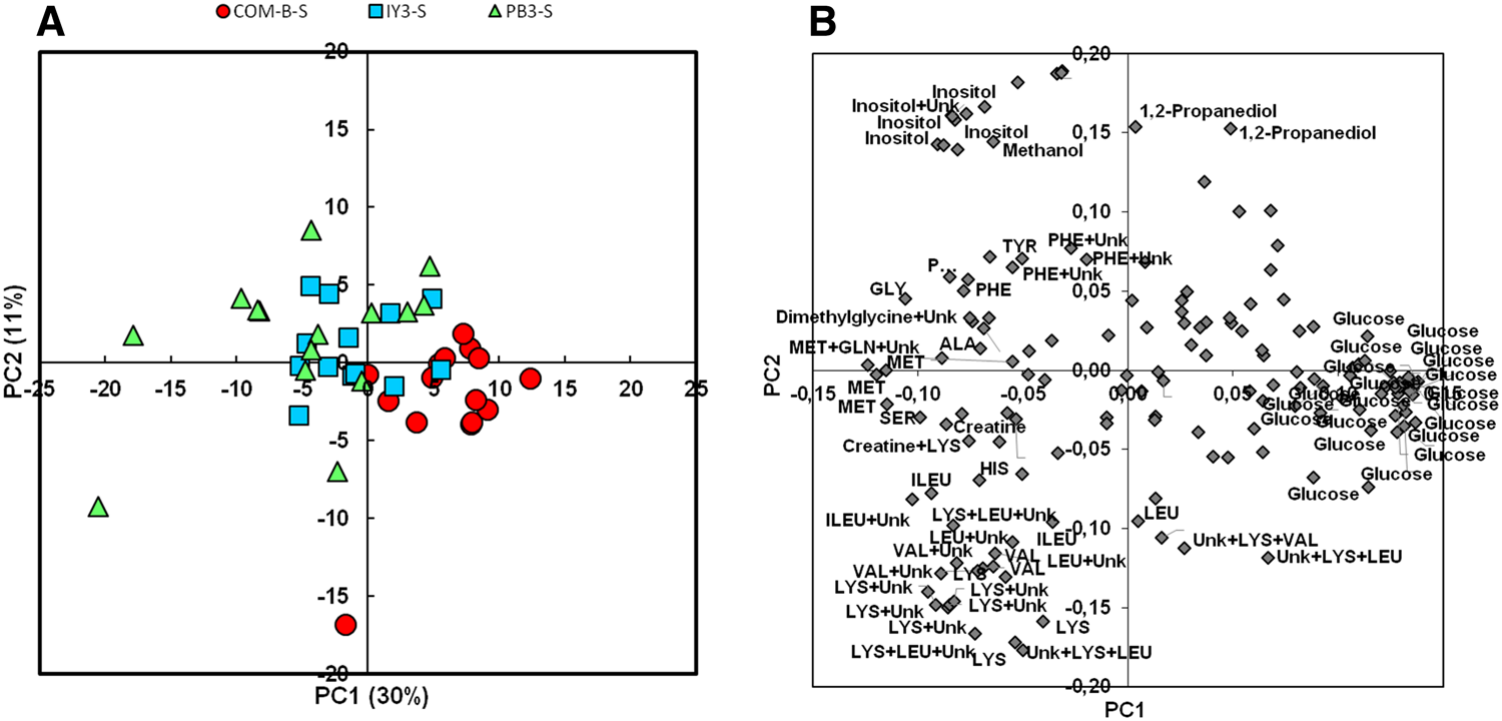
PCA of ^1^H-NMR metabolite profile in plasma of a selected line of trout fed a plant-based diet (PB3 closed green triangles), mix-supplemented with insect and yeast ingredients (IY3, squares) compared to a commercial-like diet (COM-B, circles) in Experiment B. (**A**) Scores plot on the PC1 × PC2 plan. (**B**) Loadings plot. Identified buckets with an absolute value of loading higher than 0.07 are annotated.

In Experiment B, the PCA showed that the plasma ^1^H-NMR profiles of fish from the selected line fed the PB3 plant-based diet tended to separate from those of fish fed the COM-B diet (Fig. 2A, PC1 x PC2 plan explaining 41% of total variance), and the profiles of fish fed the IY3 supplemented diet appeared to be intermediate between the profiles of fish fed the PB3 diet and those fed the COM-B diet. The differences between the COM-B diet and both plant-based diets (PB3-IY3, Fig. 2A,B) were related to glucose levels, on the one hand, and essential amino acids (LEU, VAL, ILE, LYS, MET), non-essential amino acids (SER, GLY), inositol, glycerol, dimethylglycine, creatine, methanol, on the other hand, which tended to be respectively lower and higher in fish fed the PB3-IY3 plant-based diets than in those fed the COM-B diet.

**Figure 3:**
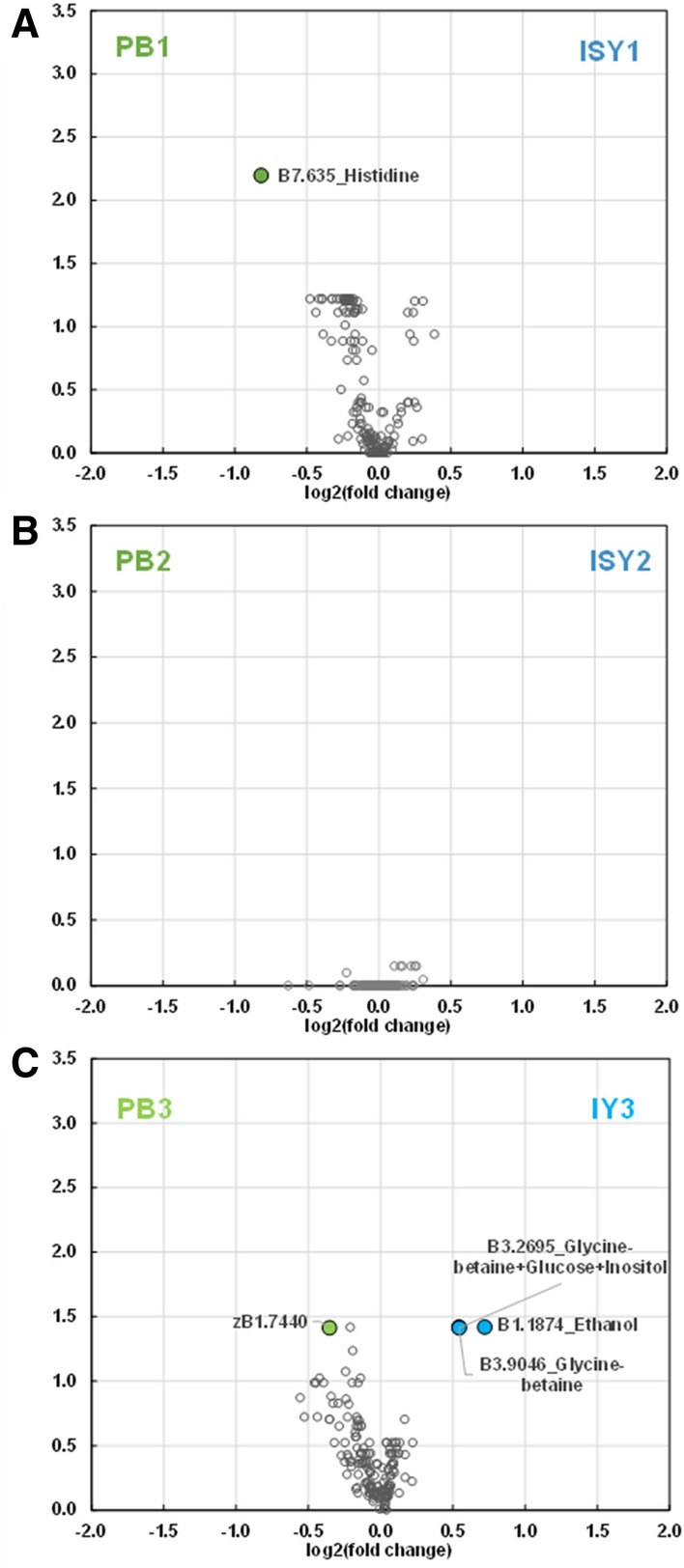
Volcano-plots of ^1^H-NMR metabolite profile in plasma of trout fed plant-based diets with or without supplementation of alternative ingredients. (**A**) Plant-based diet (PB1) and insect, spirulina and yeast mix-supplemented diet (ISY1) in Experiment A. (**B**) Plant-based diet (PB2) and insect, spirulina and yeast mix-supplemented diet (ISY2) in Experiment A. (**C**) Plant-based diet (PB3) and insect and yeast mix-supplemented diet (IY3) in Experiment B. Wilcoxon test with FDR correction (adjusted *P *< 0.05).

The ^1^H-NMR profile of fish fed each of the supplemented plant-based diets (ISY1, ISY2 or IY3) compared to the corresponding plant-based diets (PB1, PB2 or PB3) profile using volcano plots showed that there were very few discriminating compounds (Fig. 3). In Experiment A, only histidine was significantly lower in ISY1 than in PB1 (Fig. 3A). In Experiment B, glycine betaine and ethanol were significantly higher in IY3 than in PB3 and an unknown circulating lipid zB1.744 was significantly lower in IY3 than in PB3 (Fig. 3C).

### Plasma ^1^H-NMR of selected and non-selected lines of fish fed the PB supplemented diet

**Figure 4:**
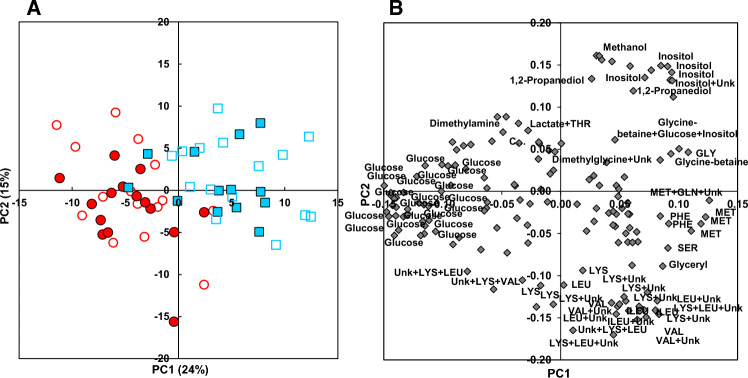
PCA of ^1^H-NMR metabolite profile in plasma of trout fed plant-based diets mix-supplemented with insect and yeast (IY3, blue squares) compared to a commercial-like diet (COM-B, red circles) in a line selected for a plant-based diet (closed symbol) and a non-selected line (open symbol) in Experiment B. (**A**) Scores plot on the PC1 × PC2 plan. (**B**) Loadings plot. Identified buckets with an absolute value of loading higher than 0.05 are annotated.

In Experiment B, a PCA indicated that the plasma ^1^H-NMR profiles of fish from the selected and non-selected lines (Fig. 4A) were neither clearly separated along PC1 (24 % of total variance) nor along PC2 (15% of total variance). However, there was a clear discrimination on PC1 in the plasma profiles of fish fed the IY3 diet compared to that of fish fed the COM-B diet in each of the lines studied. The differences between the COM-B diet and the plant-based supplemented diet (IY3) along PC1 (Fig. 4B) were related to glucose on the negative side, higher in fish fed the COM-B diet, and to essential amino acids (MET, PHE, VAL, LEU, LYS), non-essential amino acids (GLY, SER), glycine betaine, inositol, 1-2-propanediol, ethanol, methanol and glycerol on the positive side, higher in fish fed the IY3 plant-based diet. The OPLS-DA of plasma ^1^H-NMR profiles discriminating fish from the selected and non-selected lines for each diet are shown in Fig. S3 for a commercial-like diet (COM-B) or a plant-based diet supplemented with insect and yeast (ISY3).

**Figure 5:**
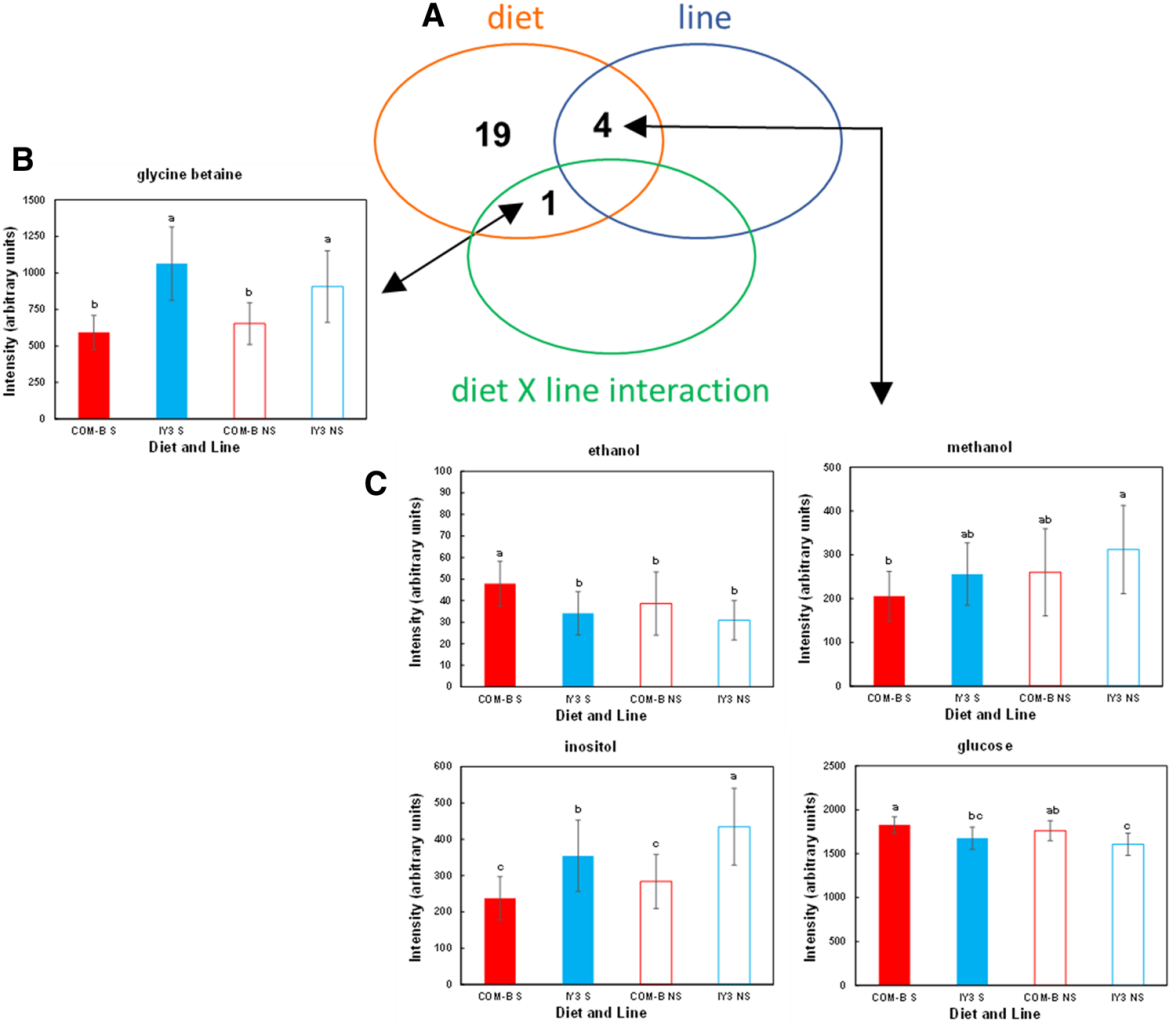
Two-way ANOVA results for the effect of diet, line and their interaction on plasma ^1^H-NMR in Experiment B. (**A**) Venn diagram summarizing the 24 annotated metabolites measured in plasma for which diet, line or their interaction had a significant impact on their relative intensity as tested by the two-way ANOVA and presented in Table 5 (*P*<0.05). (**B**,**C**) Metabolite relative intensity for the selected (S) and non-selected (NS) lines fed COM-B and IY3 diets. Mean ± SD, n = 15 fish, except for S line fed IY3 diet for which n = 13. For each metabolite, means accompanied by the same letter are not significantly different according to a Wilcoxon test with correction for multiple testing (*P* *<* *0.05*).

Two-way ANOVAs of the 37 selected spectra regions, representative of all the annotated compounds, were performed to determine the effects of diet, line, and their interaction on these annotated compounds (Table 5, *P *< 0.05 and Fig. 5). They confirmed the trends observed in the PCA analysis, showing significant differences between diets in terms of glucose, essential amino acids (ILEU, MET, PHE, LYS), non-essential amino acids (GLY, SER, ALA), glycine betaine, inositol, 1-2-propanediol, ethanol, methanol, and glycerol. They also showed significant differences in cholesterol, VLDL-LDL, unsaturated fatty acid, and other non-identified circulating lipids (zB2.0137, zB1.7440, zB1.8110, zB3.2325). The significant differences related to the line effect were observed for glucose, inositol, ethanol, and methanol. These metabolites showed different patterns corresponding to a higher difference related to diet in the selected line compared to the non-selected line for ethanol and methanol and a lower difference related to diet in the selected line compared to the non-selected line for inositol and glucose (Fig. 5C). Finally, we observed only one metabolite, namely glycine betaine, for which the relative intensity was significantly modified by the interaction between diet and line (Table 5, Fig. 5A). The relative intensity of glycine betaine in fish fed the IY3 diet was higher in the plasma of the selected line compared to the non-selected line. In fish fed the COM-B diet, the glycine betaine intensity was lower in the selected line compared to the non-selected line (Fig. 5B).

**Table 5:** Annotated spectra regions of metabolites from Experiment-B for which diet, line selection or interaction between diet and line selection had a significant effect (two-way ANOVA *P* <0.05) on its relative intensity.

Spectrum region_metabolite	Diet P-value	Line selection P-value	Diet x Line P-value
B1.0221_isoleucine*	**4.20E-02**	5.64E-01	6.65E-01
B1.1529_1,2-propanediol*	**3.35E-05**	1.10E-01	7.05-02
B1.1874_ethanol	**8.69E-04**	**4.59E-02**	3.37E-01
B1.4757_alanine*	**3.11E-02**	3.19E-01	2.98E-01
B2.6485_methionine	**9.61E-07**	9.67E-01	5.73E-01
B2.9323_dimethylglycine+Unk	**3.13E-04**	9.98E-01	3.62E-01
B3.0166_lysine*	**7.75E-03**	4.01E-01	6.74E-01
B3.3540_methanol*	**1.52E-02**	**2.38E-02**	9.24E-01
B3.4965_glucose	**6.80E-06**	**3.98E-02**	9.51E-01
B3.5627_glycine	**1.49E-04**	1.09E-01	7.14E-01
B3.9046_glycine-betaine*	**7.67E-09**	5.79E-01	**4.52E-02**
B3.9649_serine	**3.38E-02**	6.00E-01	2.02E-01
B4.0669_Inositol*	**1.29E-07**	**6.77E-03**	8.05E-01
B7.4330_phenylalanine*	**3.62E-03**	8.76E-01	1.39E-01
zB0.6680_cholesterol	**1.76E-02**	6.62E-01	6.17E-02
zB0.9210_VLDL_LDL	**2.41E-11**	1.67E-01	4.41E-01
zB1.2495_VLDL_LDL	**5.71E-03**	1.01E-01	2.22E-01
zB1.7440*	**5.16E-09**	2.71E-01	8.94E-01
zB1.8110	**2.55E-04**	1.41E-01	7.80E-01
zB2.0137	**4.23E-03**	2.53E-01	4.67E-01
zB2.7400_unsaturated_FA*	**6.83E-05**	9.28E-01	4.121E-01
zB2.8109_unsaturated_FA	**1.17E-02**	1.30E-01	1.03E-01
zB3.2325	**2.61E-03**	9.40E-01	8.58E-01
zB4.3205_Ɛ_Glyceryl	**2.13E-05**	1.65E-01	8.71E-01

Twenty-four out of the 37 selected spectra regions representing all the annotated compounds and lipid signatures in plasma of rainbow trout fed a plant-based diet supplemented with insect and yeast (IY3) ingredients compared to a commercial-like diet (COM-B) in a line selected (S) for plant-based diets and a non-selected line (NS). *P-values < 0.05* are in bold.

*Log2-transformed variables prior to ANOVA analysis.

## Discussion

This work aimed at testing the current key elements for improved growth performances of fish fed plant-based diets. This comprised the relevant choice of plant-based ingredients, the supplementation with a mix of alternative ingredients (insect hydrolysate, yeast wall, microalgae biomass) and the genetic selection for fish adaptation to plant-based diet. The results presented here clearly demonstrated that each of these elements contributed to improve growth performances compared to fish fed a control plant-based diet. However, the underlying mechanisms for improved growth performances related either to formulation of plant-based diet or to genetic selection were different. Full plant-based diets usually negatively affect the feed intake of numerous fish species due to low acceptability of plant-based ingredients^38,39^. Consequently, they display lower growth performances, while feed efficiencies remain stable or are reduced. This has been clearly demonstrated in rainbow trout fed a plant-based diet throughout its life cycle^37,40^. The incorporation of high-quality plant ingredients can restore growth performances essentially by stimulating feed intake but we have nevertheless observed variations within this known pattern. In Experiment A, the fish fed the control plant-based diet PB1 showed an increase in feed intake compared to the commercial-like diet. Such an increase in feed intake is observed in fish fed unbalanced diets^41^. Furthermore the choice of well-balanced and low-cost raw plant-based feedstuffs in PB2 diet was able to almost but restore growth performance compared to commercial diet COM-A while decreasing feed intake and maintaining feed efficiency. The better acceptability and higher digestibility of selected plant-based ingredients is sufficient enough to explain the improved growth performances of PB2. The balance in essential amino acids of the diets has however to be taken into account as the presence of amino acids sensing-systems in the brain has been demonstrated^42^. This has been connected to the effect of some amino acids on feed intake among which leucine has been demonstrated to be anorexigenic^43^. The leucine content of the plant-based diets tested was higher than that of commercial diet. Furthermore, the leucine content was especially higher in PB2 diet and this could explain the decreased feed intake observed with this diet while maintaining feed efficiency. In Experiment B, the PB3 low-cost plant-based diet did not result in the same growth performances as that of commercial diet COM-B with lower feed intake, SGR and feed efficiency, although its formulation was roughly similar to that of PB2 diet. Such discrepancies probably relate to the different conditions between the two experiments (facilities, feeding pattern, environmental parameters) as it was shown that rearing conditions can modulate the response of fish to plant-based diets^44^. This highlights the crucial importance of repeated tests to ensure robust progress in the development of sustainable fish feed.

Concerning the mix-supplemented diets, the growth performances of fish fed the three diets tested (ISY1, ISY2 and IY3) tended to reach that of fish fed commercial-like diets, demonstrating the beneficial effect of combining alternative ingredients that have already been tested separately^5,10,28–30^. It is, however, not possible to draw conclusions on the additive or synergic effect of the ingredients tested as the inclusion level of each of these ingredients was rather low (5%).

Genetically selecting fish for higher growth when fed a plant-based diet increased voluntary food intake more than final body weight, resulting in reduced feed efficiency. This confirms the genetic background for feed-acceptability of plant-based diets observed in salmonids^12,26^. A higher voluntary feed intake has also been observed in existing selected trout lines adapted to plant-based diets compared to non-selected lines^15,17,25,45^. Interestingly, we observed a highly significant interaction between selection effect and diet effect for the feed intake and a less significant interaction for growth rate. This interaction was demonstrated by the significantly higher feed intake of the selected line fed the mix-supplemented diet and the plant-based diet compared to the non-selected line, and no differences in the feed intake between lines of fish fed the commercial diet. Finally, the growth performances of fish in the selected line, fed the mix-supplemented diets, were significantly improved compared to the referent plant-based diet but did not reached that of the non-selected line fed a commercial-like diet knowing that only half of the genetic effect was tested in our experiment. This indicates however that the selected line has both the ability to adapt to a full plant-based diet and the ability to adapt to alternative ingredients.

The improved zootechnical performances of fish fed mix-supplemented diets and that of fish of the selected lines constitutes an interesting background to the analysis of the underlying metabolic processes involved in restoring fish performances by using plasma metabolomics. The plasma was collected after the post-prandial absorption of feed nutrients to study fish metabolome rather than food metabolome. We demonstrated that the fish metabolome is remarkably stable with full plant-based diets.

The metabolome of fish fed a plant-based diet compared to that of fish fed a marine diet is classically characterised by a delay in the absorption of essential amino acids^27,31^ induced by gut intolerance to plant ingredients. In the two present experiments, we confirmed that essential amino acids circulating in the plasma were maintained at high levels in fish fed plant-based diets compared to those fed commercial-like reference diets that include fish ingredients. This delay could be related to amino acid unbalance which in turn could amplify their effects on feed intake^43^. Furthermore, the plant-based diet also induced a lower circulating glucose compared to the commercial-like reference diet. The commercial-like diet also contained plant-based ingredients rich in complex carbohydrates that could induce a high circulating level of glucose in salmonids^46,47^. The asynchronous supply of energy substrates and essential amino acids results in a typical metabolic scope of fish fed plant-based diets compared to commercial-like diets. Our results clearly demonstrate that the plasma metabolomes of fish fed all plant-based diets were maintained within this metabolic scope regardless of the improvement tested, either by diet formulation or by supplementation with specific sustainable ingredients.

There were very few changes in plasma metabolomes induced by supplementation with a mix of alternative ingredients. Moreover, the metabolic effects of each of the ingredients tested are difficult to detect at such low inclusion level of 5%, and they are more discernible in tissues, such as muscle and liver, than in plasma^28,29^. The few observed changes were contingent on the plant-based diet tested. Histidine was reduced by the supplementation with a mix of insect, yeast and spirulina ingredients compared to the PB1 diet in line with changes in growth performances. The plasma level of histidine might reflect the preservation of vital cell functions such as osmolality and buffering capacity^29,30,48^. These functions could be affected by gut integrity and immunity alterations induced by plant-based diets^40^ and could be restored with alternative ingredients supplementation. These known alterations might have been reduced with the other two plant-based diets -namely PB2 and PB3- by the use of whole plant ingredients rather than processed or purified ones. In Experiment B, glycine-betaine (also named betaine) relative intensity was increased by the supplementation of a mix of insect and yeast ingredients of the third plant-based diet, PB3 in line with changes in growth performances. Levels of choline, glycine-betaine and their derived metabolites (trimethyl-glycine, serine and glycine) are regularly found to be affected in fish fed plant-based diets^31,33,35,49^. Glycine-betaine is involved in a methyl-donor function^50^, in the synthesis of phospholipids and fatty acids^50,51^ and in cell osmotic protection^52^. The observed difference in glycine-betaine could thus be related to the restoration of one of these functions by the ingredients supplied. Alternatively, glycine-betaine is a specific ingredient of yeast culture substrate and thus its accumulation in plasma could also be directly related to the supplementation of a plant-based diet with a yeast extract.

The plasma metabolome was faintly but specifically affected by the selection of fish for adaptation to plant-based diets. It was surprisingly similar in the selected line and in the non-selected line, both in fish fed a commercial-like diet (COM-B) and in fish fed a mix-supplemented diet (IY3). The lines selected for a given trait usually exhibit specific differences in the related metabolic pathways, for instance, a line selected for lipid content^53^. However, the ability to use plant-based diets is a complex trait which integrates low palatability of diets^54^, gut susceptibility to anti-nutritional factors^38,55,56^, alteration in liver function^57^, microbiota modulation related to complex carbohydrates^58,59^, and metabolic pathway adaptation to an unbalanced supply of essential nutrients such as fatty acids and amino acids^60,61^. In terms of functionality, it has been established that selected lines^17^ were able to compensate for the delay in amino acid absorption generally observed in salmonids fed plant-based feedstuffs^17,27,60,61^. We did not observe such a difference in amino acid accumulation between the selected and non-selected lines, but this might be explained by either the plasma sampling that took place later after the last meal than in other studies^17^ or by the differences between the metabolic characteristics of the selected lines^36^.

There were few but interesting discriminations in the plasma metabolome between lines of trout either non-selected or selected for their adaptation to plant-based diets. Some of these discriminations were both observed in fish fed mix-supplemented diet and commercial-like diet whereas others were observed mainly in fish fed a mix-supplemented diet. It is interesting to further explore these differences related to complementary metabolic pathways. The lines were differentiated in both the COM-B and IY3 diets by inositol and circulating lipids related to unsaturated fatty acid and lipoproteins. The changes in plasma circulating lipids are usually related to differences in fatty acid composition of the plant-based diet^41^. This suggests, however, that selected lines could adapt their lipid metabolism when fed plant-based diets although in itself this could not contribute to improved growth performances. Inositol is also highlighted in the plasma metabolome of a rainbow trout selected line compared to a non-selected one^35^. The changes in inositol are especially interesting as it is a precursor of phospholipids, and in its phosphorylated state, it induces various biological functions including insulin resistance, osmoregulation and antioxidant responses^62,63^. One less speculative explanation for the lowering of the inositol status in the selected line is that it could simply be evidence of the redesign of essential phosphatidyl-inositol in different tissues, including the brain. This could be complemented by a better oral acceptability of plant-based diets mediated by inositol^64^ and thus could lead to a better adaptation of the fish to plant-based diets by increasing their feed intake. The lines were also differentiated in both COM and IY3 diets by the status of ethanol and methanol, 48 hours after the last meal. This could be related to a microbiota adaptation to plant-based diets as it has been established that the lines of rainbow trout selected for adaptability to plant-based diets showed higher gut and microbiota tolerance to plant ingredients^35,36,45^. In addition, the microbiota could produce ethanol from carbohydrates^65^ and this production could vary with the two types of diets tested (COM-B and IY3) depending on the composition of the microbiota and the nature of the carbohydrates. The analysis of the digesta metabolome a few hours after the last meal in a selected line of rainbow trout fed a plant-based diet reveals differences in numerous metabolites of the carbohydrate metabolism^35^. Thus, the specific contribution of microbiota end-product substrates to fish metabolism needs to be further investigated by comparative analysis of post-prandial time course in digesta and plasma metabolomes. The circulating ethanol and methanol in plasma could also be of external origin due to manufacturing residues of the ingredients and the diet, but this cannot account for a line effect. Alternatively, it could be an end product of fish energy metabolism, but endogenous ethanol is only produced in a few species of fish enduring extreme anaerobic conditions^66^.

Line-specific differences related within fish fed the mix-supplemented diet IY3 were observed for plasma glucose and glycine-betaine that were higher in the selected line than in the non-selected one. This was associated with an increased feed intake together with a decreased feed efficiency. The higher glucose in the selected line fed a mix-supplemented diet mimics the accumulation of glucose observed in fish fed COM diets, as previously discussed. Together with higher glutamine and alanine found in the selected line fed COM diets, this suggests that the selected line could better adapt its energy metabolism than the non-selected one. This confirms the specific metabolic adaptation of selected lines of rainbow trout fed plant-based diets^16^. The other differences observed for glycine betaine and related compounds in the plasma metabolome of the selected line fed a supplemented plant-based diet could be associated with yeast supplementation^28^. This demonstrates for the first time that selected lines can not only adapt their lipid and energy metabolism to the better utilisation of plant-based diets, but also that they could adapt their metabolism to alternative ingredients complementing the plant-based diets.

This work explored for the first time three complementary strategies to adapt rainbow trout nutrition to sustainable fish feeds: alternative formulation of plant-based diets with raw feedstuffs, the supplementation of plant-based diets with a mix of alternative ingredients (insect, microalgae, yeast), and the adaptation of fish to plant-based diets by genetic selection. These different strategies were successful in improving the growth performances of fish fed plant-based diets, although not always up to the levels of fish fed a commercial-like diet. The study of the plasma metabolome revealed that the alternative formulation of a plant-based diet and its supplementation with alternative ingredients could help suppress the known alteration of fish metabolism due to gut alteration in different ways. This argues for a systematic analysis of the plasma metabolome in sustainable fish-feed development.

## Material and methods

### Origin of fish

Two experiments were conducted in two different INRAE experimental facilities, namely, NUMEA Donzacq, France, https://doi.org/10.15454/GPYD-AM38 and PEIMA Sizun, France, https://doi.org/10.15454/1.5572329612068406E12. In the first experiment (Experiment A), rainbow trout were taken from an INRAE synthetic line. Synthetic line has been created in the 1980s through mixing of fish from different French farms populations, and a few from Denmark and USA to get a large genetic basis. It was then closed and maintained in our INRAE experimental facilities over generations with large number of breeders and without artificial selection to maintain genetic variability. The fish of this synthetic line were maintained and produced at the INRAE experimental hatchery (NUMEA Lées-Athas, France, https://doi.org/10.15454/GPYD-AM38). The fish of this synthetic line were transferred at the fry stage to the INRAE experimental facility at NUMEA Donzacq. In the second experiment (Experiment B), rainbow trout from an INRAE line, selected for its ability to survive and grow on a full plant-based diet for four generations^15^, was used to be compared with the INRAE synthetic non-selected line. These lines were maintained and reared at the INRAE experimental hatchery of PEIMA (Sizun, France). The mating design is presented in Fig. S2. Briefly, eggs laid by 30 females of the INRAE-autumnal line on the same day were combined and divided into two groups. One group was fertilised with individually frozen sperm from 27 fourth generation males in the selected line (**selected**). The other group was fertilised with individually frozen sperm from 31 males in the synthetic line (**non-selected**). The comparison between the selected line and the non-selected line thus corresponds to the half-response of the expected genetic selection since it only relates to the male pathway.

### Diets

The diet designs used in each of the two experiments are summarised in Fig. S1. In Experiment A, five diets were formulated by Le Gouessant (Lamballe-Armor, France) and produced at the INRAE feed facility NUMEA (Donzacq, France): (a) a referent commercial-like diet based on fish meal and fish oil (COM-A), (b) two different full plant-based diets (PB1) and (PB2) based on vegetable oil and plant ingredients, and (c) the same two plant-based diets supplemented with a mix of 5% insect hydrolysate extract, 5% spirulina meal and 5% yeast cell wall extract (ISY1) and (ISY2) (Table 1). Briefly, the difference between PB1 and PB2 is as follows: plant ingredients in the PB1 diet match the processed and semi-purified protein ingredients traditionally used for scientific purposes, while those selected for the PB2 diet were their less processed and non-purified low-cost counterparts (whole seeds, raw feedstuffs, and by-products). The ingredients tested were produced by COPALIS Industry (Boulogne-sur-Mer, France) for insect *Tenebrio molitor* larvae protein hydrolysate, by Spirulina solutions (www.spirulinasolutions.fr) for dried biomass of spirulina *Arthrospira platensis* cultivated in autotrophic conditions, and by Lesaffre Industry (Marcq-en-Baroeul, France) for yeast *Saccharomyces cerevisiae* cell wall extract .

In Experiment B, three diets were formulated and produced by Le Gouessant (Lamballe-Armor, France): (a) a commercial-like diet (COM-B), (b) a plant-based diet (PB3) similar to the low-cost diet of the first experiment and (c) the same plant-based diet supplemented with 5% insect hydrolysate extract and 5% yeast wall extract (IY3) (Table 2).

The comparative formulations of the diets used in Experiments A and B are presented Table S1. The diets were formulated to be iso-proteic, iso-lipidic and iso-energetic in each experiment. Amino acid composition, fibre and carbohydrate contents of the experimental diets are presented in Tables S2, S3.

### Ethical approval

Fish care and sampling were undertaken in accordance with the European Directive 2010/63/EU and French legislation for animal welfare used for scientific purpose (National Guidelines for Animal Care, Decree No. 2013–118, February 1, 2013). Growth experiments, as well as fish manipulation and fish euthanasia were performed in accordance with the guidelines reviewed and the study was approved by ethical committees Ethical Committee Aquitaine Fish and Birds CEEA – 073 and Finisterian Ethical Committee in Animal Experiments CEEA - 074 (CEFEA) (CEEA – 073 INRA 2002-36, April 14, 2002, for NUMEA facilities and CEEA-073 – CEEA-074 for PEIMA facilities). The experimental facilities and each of the scientists and staff involved in experimental manipulation and euthanasia had their own agreements (A40-2281 and B64-10-003 respectively for the NUMEA facility and N° D29–277‑02 and A64-495-1 for the PEIMA facility). This study was carried out in compliance with the ARRIVE 2.0 guidelines. The fish were anaesthetised with benzocaine (10 mg/L) in Experiment A and with tricaine methylsulfonateMS-222(0.05 g/L) in Experiment B. The fish were then euthanised by a sharp blow to the head and bled in water by sectioning their gill arches.

### Fish experiments

In Experiment A, juvenile fish (initial body weight 49.1 g ± 1.2) were randomly distributed among 15 tanks (n = 30 fish per 100-L tank), representing each of the five diets in triplicates. The initial body weights were not significantly different between the tanks (ANOVA, F = 0.14, *P *= 0.96).The fish were reared in a flow-through system at natural photoperiod and constant oxygen (> 9.0 mg/L) at a temperature of 17 °C ± 1 °C. Fish were fed twice daily manually to satiation until first unfed pellets appeared, for 64 days and corresponding feed distribution was recorded. The whole tank biomass was weighed every 3 weeks after starving the fish for 24 hours. Total feed distribution and tank biomass were recorded during the entire experiment.

In Experiment B, juvenile fish, half of which were taken from the non-selected line (initial weight 64.5 g ± 1.0) the other half of which were taken from the selected line (initial weight 69.2 g ± 1.0), were distributed among 18 tanks (n= 350 fish per 1800-L tank), representing each of the three diets in each of the two lines in triplicates. The initial body weights were not significantly different between the tanks within each fish line (Two way ANOVA F=1.30 *P=0.31*). The fish were reared in a flow-through system at natural photoperiod, constant oxygen (7.5 to 9.5 mg/L) and ambient temperature (12 °C to 18 °C). Fish were fed automatically to satiation twice daily for 85 days and the unfed pellets were collected and recorded to adjust the next day feed distribution. This latter feeding method used was more adapted to the size of the tank used. The whole tank biomass was weighed every 3 weeks after starving the fish for 24 hours. Total feed distribution and tank biomass were recorded during the entire experiment.

Voluntary feed intake (amount of feed distributed per day), specific growth rate (Ln(final body weight) – Ln(initial body weight))/number of days)) and feed conversion ratio (feed distributed/body weight gain) in each tank were calculated.

### Fish sampling

Nine fish per treatment for Experiment A and 15 fish per treatment for Experiment B were sampled 48 hours after the last meal. In Experiment B, the sampling design was very heavy as it comprised not only blood sampling for metabolomics but also a comprehensive assessment of phenotypical and quality traits together with tissue sampling for biochemical analysis. It was not possible to include all the treatments. Thus the sampling was focused on one hand on the three diets only in the selected line and on the other hand on the mix-supplemented diet IY3 and the reference diet COM-B in both the selected line and the non-selected line, according to a Latin-square design. The fish were anaesthetised with benzocaine (10 mg/L) in Experiment A and with tricaine methylsulfonate MS-222 (0.05 g/L) in Experiment B. The blood was collected, centrifuged and the plasma was collected, snap-frozen in liquid nitrogen, and stored at − 24°C until the end of the sampling procedure, then stored at – 80 °C until analysis. The fish were then euthanised by a blunt blow on the head and bled in water by sectioning their gill arches.

### Plasma analysis

Plasma samples were prepared as described previously^28^. Briefly, plasma samples were first individually thawed, then diluted with D_2_O, the diluted plasma was then transferred to a 5-mm NMR tube for NMR acquisition, which was performed immediately after each sample preparation.

One-dimensional ^1^H-NMR acquisitions (1D ^1^H-NMR) were undertaken at the INRAE Metabolome platform (https://doi.org/10.15454/1.5572412770331912E12) using parameters previously described^28^. Briefly, for plasma, one-dimensional Carr-Purcell-Meiboom-Gill (CPMG) ^1^H-NMR spectra with water presaturation and a 90° pulse angle were acquired. In addition, a simple water presaturation pulse sequence (zgpr) was used to analyse circulating lipoproteins and the two corresponding datasets were combined as previously described^67^.

The free induction decays and spectra were processed using the NMRProcFlow tool (nmrprocflow.org)^68^. The spectra regions stemming from intelligent bucketing were named according to the centre of the corresponding region (e.g. B4.2670 for a bucket centred at 4.2670 ppm). If they met the criterion of a signal-to-noise ratio greater than 3, their area was integrated and standardised using constant sum normalisation (CSN). The assignments in the 1D ^1^H-NMR spectra were based on previous studies^28^, an in-house database, a public database (BMRB Metabolomics, https://bmrb.io/metabolomics/) and the ChenomX NMR Suite library 8.3 (ChenomX Inc., Edmonton, Canada). When annotated, each integrated spectral region was also named after the metabolite it corresponded to. A set of 37 annotated compounds (Table S4) was constituted, using the most representative spectra region for each metabolite identified to undertake interaction analyses between lines and diet in Experiment B.

### Statistics

Univariate analyses were performed using the BioStatFlow tool based on R scripts (v2.9, http://www.biostatflow.org)^69^. Tank effect on fish initial body weight was tested using one-way ANOVA (*P *< 0.05). Dietary and/or line effects on fish performances were tested via variance analyses (ANOVA, one-way and two-way, with interactions when necessary, and a significance threshold of *P *< 0.05) to summarise most of the variance induced at tank level (N = 3). Normal distribution of the dependant variables was confirmed using the Shapiro-Wilk test (*P *> *0.05)* at tank level. Multiple comparison Tukey’s tests were implemented to test for differences (*P < 0.05*) when ANOVA was significant.

Plasma datasets of metabolomic profiles were mean-centred and scaled to unit variance prior to multivariate analyses using the BioStatFlow tool (v2.9, http://www.biostatflow.org)^69^. Principal component analyses (PCA) were used to assess the main sources of variance on metabolite profiles. These PCA were performed on fish fed the five diets in Experiment A, on fish from the selected line fed the three diets in Experiment B and on fish from both selected and non-selected lines fed the COM-B and IY3 diets in Experiment B. Partial least-squares discriminant analyses (OPLS-DA) were also performed to discriminate the differences in plasma metabolome related to fish line effect in fish fed the COM-B and IY3 diets.

The effects of the relative intensities of mix-supplemented diets on metabolites were compared to their respective plant-based diets on a volcano plot. Differences in individual metabolites were tested using the Wilcoxon test with *P*-values adjusted for multiple testing (false discovery rate using Benjamini Hochberg correction). A 1.2 log fold change threshold between means and a *P*-adjusted value < 0.05 were considered significant.

Two-way ANOVAs were performed on 37 annotated spectra regions of plasma (Table S4) of the selected and non-selected lines of fish fed the mix-supplemented diet (IY3) and the commercial-like diet (COM-B) to assess the relative effects of diet, selection and the interaction between diet and selection. Prior to this, the normal distribution of the relative intensity of each metabolite was assessed using the Shapiro-Wilk test (*P > 0.05* for 24 out of 37 annotated spectra regions) and, when necessary, the metabolite relative intensity was log2-transformed to correct the distribution (13 out of 37 annotated spectra regions). A Wilcoxon test was performed to assess pair-pair differences when ANOVA was significant.

### Supplementary Information

Supplementary Information.

## Data Availability

The metabolomic data that support the findings of this study are openly available on request in the Recherche Data Gouv repository at, https://doi.org/10.57745/XYUDUT. Data will be available from corresponding author on reasonable request.
