# Supplementary material for: New alternative ingredients and genetic selection are the next game changers in rainbow trout nutrition: a metabolomics appraisal
Source: Sci Rep. 2023 Nov 10;13:19634. doi: 10.1038/s41598-023-46809-2 (PMC10638236; doi:10.1038/s41598-023-46809-2)
Supplement: Supplementary file 1 — Supplementary Information. [file 41598_2023_46809_MOESM1_ESM.docx]

**Supplemental information**

**Title:** **New alternative ingredients and genetic selection are the next game changers in rainbow trout nutrition: a metabolomics appraisal.**

**Authors:** Simon Roques, Catherine Deborde, Sandrine Skiba-Cassy, Francoise Médale, Mathilde Dupond-Nivet , Florence Lefevre, Jerôme Bugeon, Laurent Labbé, Yann Marchand, Annick Moing, Benoit Fauconneau

**Table S1** Comparison of protein and lipid ingredients of the experimental diets designed for the two experiments *i.e.* commercial (COM-A and COM-B) and plant-based (PB1, PB2 and PB3) diets for rainbow trout feeding.

| Ingredients (g.100g^-1^ FW) | COM-A | COM-B | PB1 | PB2 | PB3 |
| --- | --- | --- | --- | --- | --- |
| Fish meal | 21.0 | 25.6 |  |  |  |
| Fish oil | 4.9 | 6.0 |  |  |  |
| DHA-rich algae meal |  |  | 6.84 | 6.84 | 6.84 |
| Rapeseed oil | 14.65 | 13.0 | 17.05 | 16.65 | 15.6 |
| Linseed oil |  |  | 1.05 | 1.1 | 1.0 |
| Wheat gluten hydrolysed |  | 2.0 | 3.0 |  |  |
| Corn gluten |  | 10.0 |  | 19.2 | 19.0 |
| Pea protein |  |  | 15.0 | 13.55 | 13.5 |
| Faba bean protein |  |  | 20.00 |  |  |
| Soy concentrate | 5.0 | 5.0 | 16.7 |  |  |
| Soybean meal | 6.5 | 12.0 |  | 14.3 | 14.00 |
| Rapeseed meal | 3.0 | 1.0 |  |  |  |
| Guar meal |  |  |  | 8.5 | 8.5 |
| Peeled faba bean | 18.0 | 17.5 |  |  | 14.8 |
| Whole wheat | 10.2 | 5.0 | 13.6 |  | 2.7 |
| Feather meal proteins | 5.00 |  |  |  |  |
| Blood product | 3.00 |  |  |  |  |
| Poultry meal | 7.00 |  |  |  |  |

**Table S2** Amino acid composition of the experimental diets (g.100g^-1^ FW)

|  | COMA | PB1 | PB2 | ISY1 | ISY2 | COMB | PB3 | IY3 |
| --- | --- | --- | --- | --- | --- | --- | --- | --- |
| Lysine | 2.8 | 2.7 | 2.7 | 2.7 | 2.7 | 2.8 | 2.6 | 2.6 |
| Méthionine | 1.1 | 1.1 | 1.1 | 1.1 | 1.1 | 1.1 | 1.1 | 1.1 |
| Méthionine + Cystine | 1.7 | 1.6 | 1.5 | 1.6 | 1.6 | 1.7 | 1.5 | 1.6 |
| Histidine | 1.0 | 0.9 | 0.9 | 0.9 | 0.9 | 1.0 | 0.9 | 0.9 |
| Arginine | 2.5 | 2.9 | 2.7 | 2.7 | 2.4 |  |  |  |
| Thréonine | 1.8 | 1.6 | 1.6 | 1.7 | 1.7 | 1.8 | 1.6 | 1.7 |
| Tryptophane | 0.4 | 0.4 | 0.4 | 0.4 | 0.3 | 0.4 | 0.4 | 0.4 |
| Leucine | 0.9 | 2.1 | 3.9 | 2.0 | 3.6 |  |  |  |
| Isoleucine | 1.5 | 1.7 | 1.6 | 1.8 | 1.7 |  |  |  |
| Valine | 2.0 | 1.9 | 1.8 | 1.8 | 1.8 |  |  |  |

**Table S3** Fibre and carbohydrate content of experimental diets (g.100g^-1^ FW)

|  | COMA | PB1 | PB2 | ISY1 | ISY2 | COMB | PB3 | IY3 |
| --- | --- | --- | --- | --- | --- | --- | --- | --- |
| Raw cellulose | 2.4 | 1.6 | 2.5 | 2.1 | 2.5 | 2.4 | 2.5 | 2.9 |
| Starch | 13.4 | 11.3 | 10.0 | 10.0 | 10.0 | 13.1 | 9.6 | 10.0 |
| acid detergent fibre | 2.5 | 1.9 | 2.4 | 1.6 | 1.8 | 2.5 | 2.4 | 2.0 |
| neutral detergent fibre | 5.5 | 4.4 | 5.7 | 4.0 | 4.9 | 5.5 | 5.7 | 5.3 |
| acid detergent lignin | 0.5 | 0.3 | 0.3 | 0.3 | 0.3 | 0.5 | 0.3 | 0.3 |

**Table S4** Selected annotated spectra regions of the plasma spectra of rainbow trout in experiments A and B.

| **Spectra region** | **Annotation** | **MSI Status*** |
| --- | --- | --- |
| B0.9663 | Leucine+Unknown | 3 |
| B1.0221 | Isoleucine | 2 |
| B1.0518 | Valine | 2 |
| B1.1529 | 1,2-Propanediol | 2 |
| B1.1874 | Ethanol | 1 |
| B1.4757 | Alanine | 2 |
| B2.3745 | Succinate | 2 |
| B2.4526 | Glutamine | 2 |
| B2.6485 | Methionine | 2 |
| B2.7435 | Dimethylamine | 3 |
| B2.9323 | Dimethylglycine+Unknown | 4 |
| B3.0166 | Lysine | 2 |
| B3.2065 | Choline | 2 |
| B3.3540 | Methanol | 2 |
| B3.4965 | Glucose | 1 |
| B3.5627 | Glycine | 2 |
| B3.9046 | Glycine-Betaine | 2 |
| B3.9350 | Creatine | 2 |
| B3.9649 | Serine | 2 |
| B4.0669 | Inositol | 2 |
| B4.1185 | Lactate | 2 |
| B7.0560 | Histidine | 2 |
| B7.1975 | Tyrosine | 2 |
| B7.4330 | Phenylalanine | 2 |
| zB0.6680 | Cholesterol | 2 |
| zB0.8450 | HDL | 2 |
| zB0.9210 | VLDL-LDL | 2 |
| zB1.2495 | VLDL-LDL | 2 |
| zB1.5640 | lipid mainly VLDL | 2 |
| zB1.7440 | unkown lipid signal | 4 |
| zB1.8110 | unkown lipid signal | 4 |
| zB2.0137 | unkown lipid signal | 4 |
| zB2.7400 | unsaturated_FA | 3 |
| zB2.8109 | unsaturated_FA | 3 |
| zB3.2325 | unkown lipid signal | 4 |
| zB4.3205 | Glyceryl | 3 |
| zB5.3321 | unsaturated_FA+Glyceryl | 3 |

FA, fatty acid; * MSI Status: MSI level of identification according to Sumner et al. 2007

**Figure S1** Diagram of the experimental design of Experiment-A and -B.


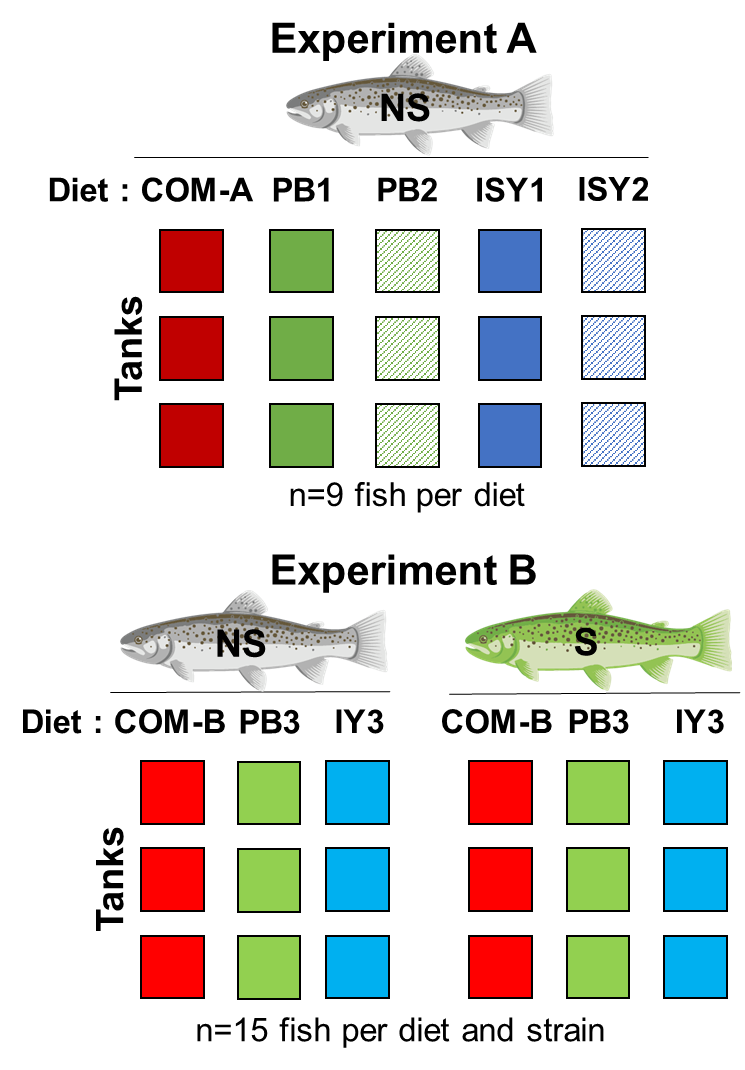


**Figure S2** Mating design of selected and non-selected lines of fish in the Experiment B

**
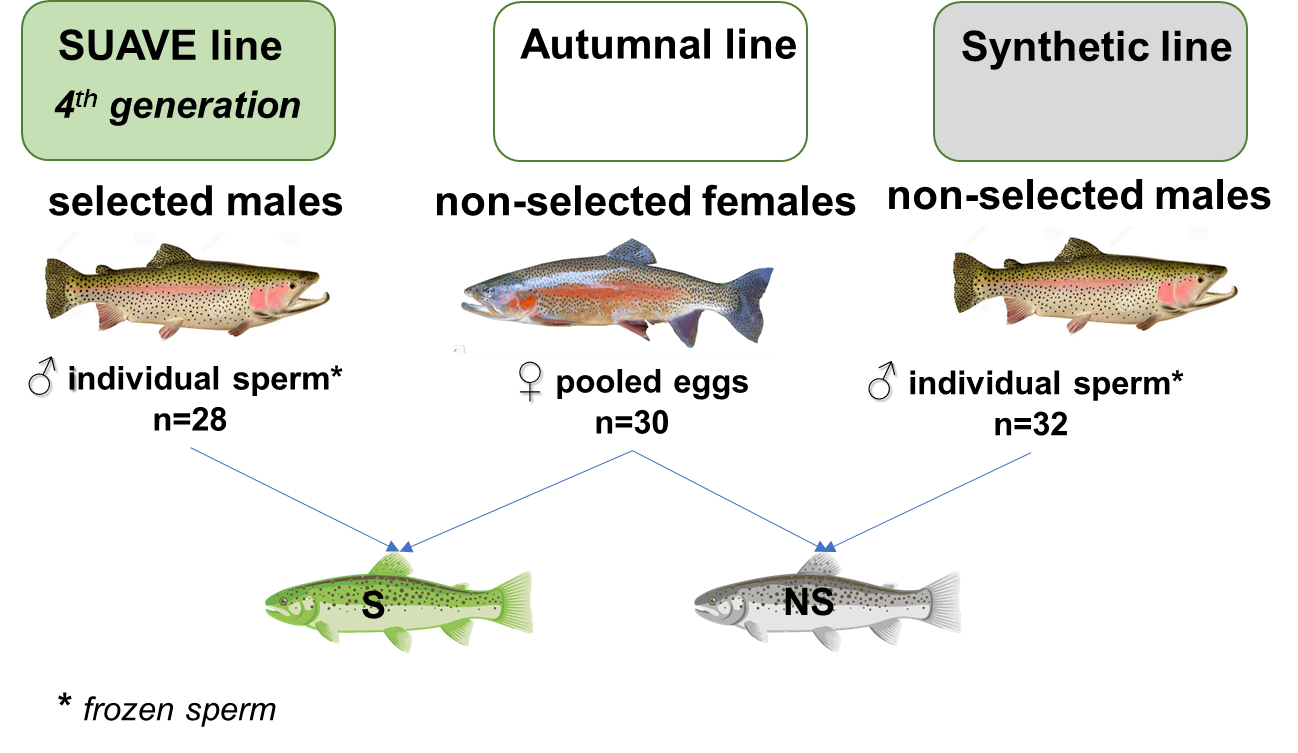
**

**Figure S3** OPLS-DA of ^1^H-NMR profiles of plasma in a selected line (S, close circle and close square) and a non-selected line (NS, open circle and open square) of trout fed either a commercial-like diet (COM-B, **A-B**) or a plant-based diet supplemented with insect and yeast (ISY3, **C-D**) in Experiment B. (A, C) Scores plot on the LV1 x LV2 plan. (B, D) Loadings plot on the LV1 x LV2 plan. Identified buckets with an absolute value of loading higher than 0.05 are annotated.

**A**

**B**

**C**

**D**

**Figure S4 Graphical abstract**


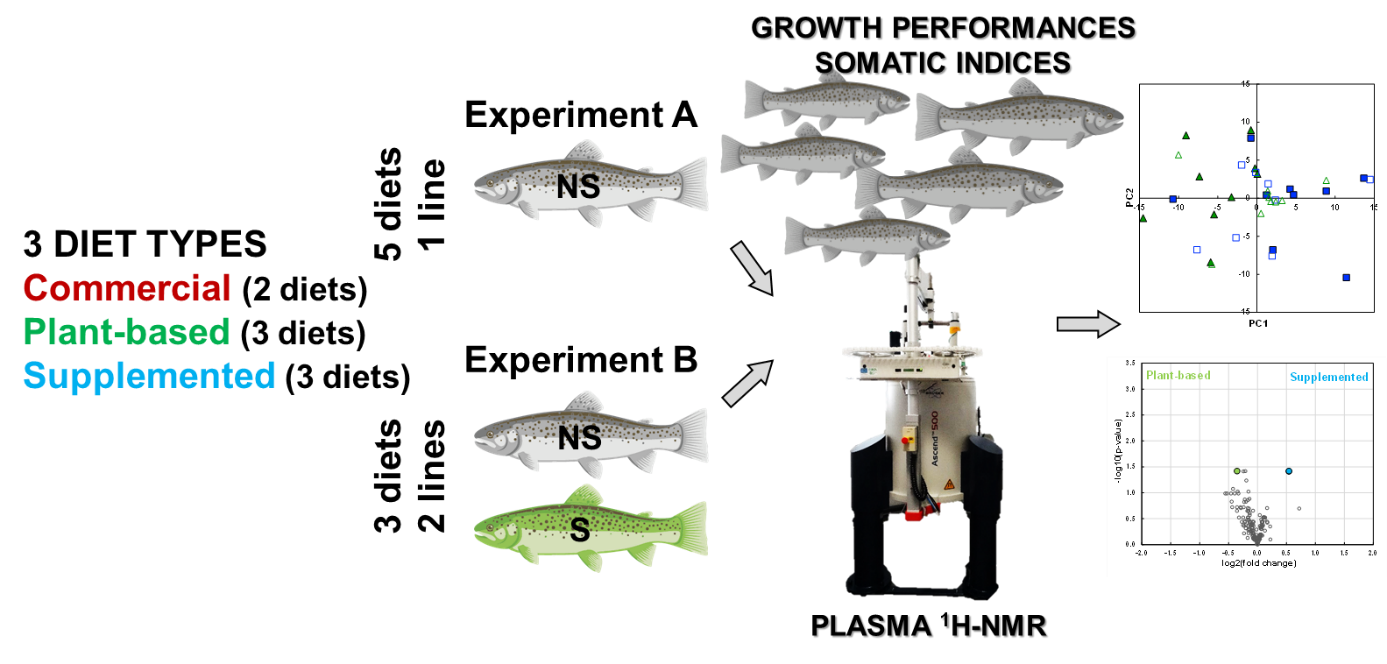


Reference:

Sumner LW., Amberg A., Barrett D., Beale MH., Beger R., Daykin CA., Fan T WM., Fiehn O., Goodacre R., Griffin J. L., Hankemeier T., Hardy N., Harnly J., Higashi R., Kopka J., Lane AN., Lindon JC., Marriott P., Nicholls AW., Reily MD., Thaden JJ., Viant MR. (2007). Proposed minimum reporting standards for chemical analysis: Chemical Analysis Working Group (CAWG) Metabolomics Standards Initiative (MSI). Metabolomics, **2007,** 3(3), 211-221. https://doi.org/10.1007/s11306-007-0082-2


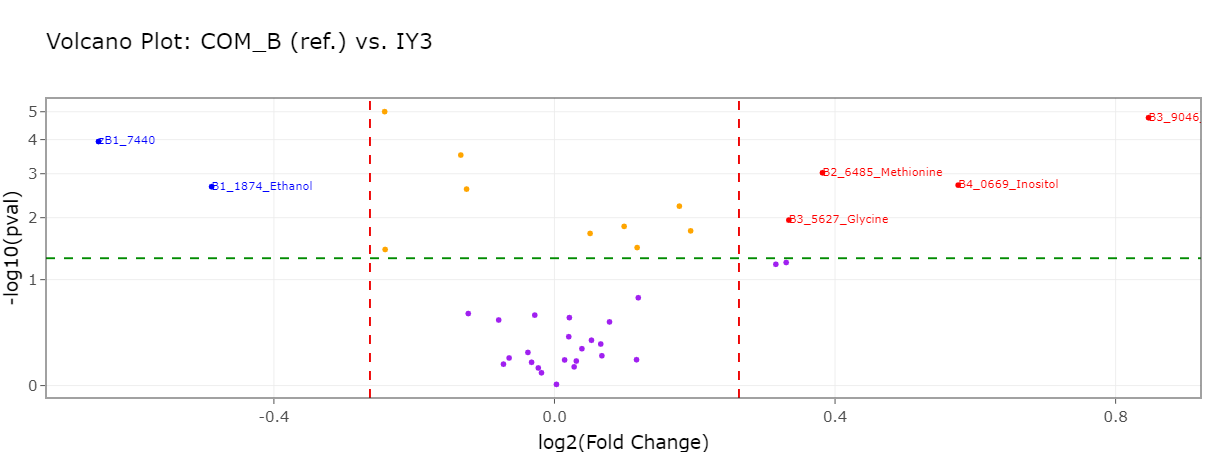


S
